# Supplementary material for: Parental and Pandemic Burnout, Internalizing Symptoms, and Parent-Adolescent Relationships: A Network Analysis
Source: J Psychopathol Behav Assess. 2023 Mar 14;45(2):428–43. doi: 10.1007/s10862-023-10036-w (PMC10010964; doi:10.1007/s10862-023-10036-w)
Supplement: Supplementary file 1 — Supplementary Material 1 [file 10862_2023_10036_MOESM1_ESM.docx]

**Parental and pandemic burnout, internalizing symptoms, and parent-adolescent relationships: A network analysis**

**Abstract**

The COVID-19 pandemic and preventive measures undertaken by many governments have had a significant impact on family relationships, which could result in worsened parenting. In our study, we used network analysis to examine the dynamic system of parental and pandemic burnout, depression, anxiety, and three dimensions of relationship with an adolescent: connectedness, shared activities, and hostility. Parents (*N* = 374; *M*_age_ = 42.9) of at least one child at the age of adolescence completed an online survey. The central symptoms in the network were parental emotional exhaustion and parental anxiety. Parental emotional exhaustion correlated negatively with activities shared with the adolescent, but positively with hostility. Anxiety correlated positively with parental emotional exhaustion. Emotional exhaustion and anxiety were the strongest bridge symptoms between parental burnout, internalizing symptoms, and parenting. Our results suggest that psychological interventions supporting parent-adolescent relationships should address primarily parental emotional exhaustion and anxiety.

**Keywords:** parental burnout; depression; anxiety; parent-adolescent relationships; network analysis

**Parental and pandemic burnout, internalizing symptoms, and parent-adolescent relationships: A network analysis**

The COVID-19 pandemic and preventive measures undertaken by many governments have had a detrimental impact on people’s daily life (Necho et al., 2021). Family relationships were one of the areas most affected by the pandemic (Feinberg et al., 2022). Remote learning for children, uncertainty regarding work and income, fear of the virus, and health concerns put parents at particularly high risk during the pandemic (van Bakel et al., 2022). The Family Stress Model (Masarik & Conger, 2017) posits that various environmental stressors causing personal distress in parents may strain family relationships and disrupt parenting, eventually threatening the health and well-being of children living in the home. Thus, stress related to the pandemic could result in worsened parenting.

During the lockdown, child emotional neglect was three times higher compared to the period before the lockdown (Vermuelen et al., 2022). One of the risk factors of child maltreatment during the pandemic could be parental burnout (Mikolajczak et al., 2018). Parental burnout is defined as “a state of intense exhaustion related to one’s parental role, in which one becomes emotionally detached from one’s children and doubtful of one’s capacity to be a good parent” (Mikolajczak, Gross, & Roskam, 2019, p. 1319). Longitudinal studies indicated that parental burnout increased during the pandemic (Aguiar et al., 2021). This was the case in most countries, particularly in those with stricter norms regarding parenthood and stricter governmental measures to prevent the spread of the virus (van Bakel et al., 2022). Apart from parental burnout, parents also suffered from increased internalizing problems (depression and anxiety), pandemic-related stress, and pandemic burnout during the pandemic (Johnson et al., 2021; Yıldırım & Solmaz, 2022). These symptoms are positively and mutually related (Skjerdingstad et al., 2021) and could collectively affect parenting, which is important due to the findings showing that positive parenting and support decreased during the pandemic (Donker et al., 2021; Vacaru et al., 2022).

In our study, we used network analysis to investigate the dynamic and mutual associations between parental burnout, pandemic burnout, parental internalizing problems (anxiety and depression), and parent-adolescent relationships. Our interest was in detecting central symptoms which could activate other symptoms or behaviors indicating parental mental health or parent-adolescents relationships (Jones et al., 2019). We focused on the latter due to the highly turbulent characteristics of this period (Branje et al., 2012), significant changes in parent-child relationships during adolescence (Keijsers et al., 2009), and the high burden the pandemic put on adolescents (Stavridou et al., 2021).

**Parental burnout**

Parental burnout is a specific syndrome resulting from enduring exposure to chronic parenting stress (Mikolajczak et al., 2018). This syndrome has received increasing attention in recent years (Mikolajczak & Roskam, 2020), and mostly during the challenging period of the pandemic (van Bakel et al., 2022). Parental burnout is characterized by a chronic imbalance of risk and resources during parenting (Mikolajczak & Roskam, 2018), which leads to three hallmark symptoms: (a) emotional exhaustion related to parenting, (b) emotional distancing from one’s children, and (c) loss of parental fulfillment (Mikolajczak & Roskam, 2020).

The prevalence of parental burnout reaches 5% (Roskam et al., 2018), but recent studies have shown that it increased during the pandemic in most countries (van Bakel et al., 2022). Parental burnout has implications for parents (e.g., escape and suicidal ideation, addictions, and sleep problems), relationships between parents (e.g., conflict, partner estrangement), and relationships between parents and children (e.g., violence and neglect; Mikolajczak et al., 2018, 2019). Burnout parents also are frequently depressed (Skjerdingstad et al., 2021) which may impact their mental health (e.g., sleep problems, addiction) but rather not their relationships with children (Mikolajczak et al., 2020).

**Parental burnout in the context of mental health consequences of the pandemic**

The COVID-19 pandemic caused severe psychological distress in the general population (Arora et al., 2022). Pandemic-related stress was not only severe but also chronic (Somma et al., 2021). In response to such prolonged stress, some people developed pandemic burnout (Yıldırım & Solmaz, 2022). Similar to job burnout (Maslach & Leitner, 2016) and parental burnout (Mikolajczak & Roskam, 2020), pandemic burnout consists of overwhelming exhaustion, feelings of cynicism and detachment from activities, a sense of ineffectiveness, and lack of accomplishment (Maslach & Leitner, 2016). Pandemic burnout also was associated with higher depression and anxiety ([hidden for blind review]). These internalizing symptoms as consequences of pandemic stress were highly prevalent in response to the pandemic, with 28% and 22% of clinically relevant anxiety and depression in the general population (Arora et al., 2022). Moreover, between 25% and 35% of parents reported clinically relevant levels of depression and anxiety, respectively (Johnson et al., 2021a), while parental stress was associated with depression (Johnson et al., 2021b). Being a mother, living with more than one child, previous psychiatric diagnosis, burnout, and anger toward the child were predictors of greater parental stress during the pandemic (Johnson et al., 2021a).

Parental burnout correlated with higher depression and stress experienced by parents during the pandemic (Prikhidko et al., 2020). A recent network analysis indicated that rumination, worry, and feeling worthless were mechanisms linking depression and parental burnout during the pandemic (Skjerdingstad et al., 2021). However, concerns about COVID-19 were associated only with the exhaustion of parental burnout (Prikhidko et al., 2020). Among parents, self-reported psychological impacts of COVID-19 were associated with higher parental burnout that, in turn, was correlated with greater increases in children’s stress behaviors (Kerr et al., 2021). Mental health conditions predicted higher parental burnout during the pandemic (Skjerdingstad et al., 2021).

**Parent-child relationships during adolescence**

Parent-child relationships typically change during adolescence (Branje et al., 2012). The main changes consist of more egalitarian relationships between parent and adolescent, increased conflict (Branje et al., 2012), decreased parental support (De Goede et al., 2009), and lower parental control (Keijsers et al., 2009). These changes indicate that adolescence is a turbulent period for adolescents (Sawyer, Azzopardi, Wickremarathne, & Patton, 2018), and their families (Finkenauer et al., 2019). The turbulent period of adolescence puts parent-adolescent relationships at risk of worsening (Donker et al., 2021).

The characteristics of adolescence suggest that during the stressful time of the pandemic, adolescents were at even higher risk of worsened relationships with their parents. For example, while parents supported adolescents less and treated them in a more egalitarian way, they may not notice adolescents’ problems related to remote learning (Wang et al., 2021). During the pandemic, higher quality of family functioning, but not peer connectedness, predicted fewer adolescent depressive symptoms (Vacaru, Beijers, & de Weerth, 2022). The quality of communication between parents and adolescents reduces posttraumatic symptomatology in children and promotes the development of posttraumatic growth (Zhen, Yao, & Zhou, 2022). During the pandemic, longitudinal studies showed decreases in positive parenting and support, but also in negative interactions between parents and adolescents (Donker et al., 2021). However, pandemic stress did not correlate with these decreases. Stress experienced by parents due to environmental factors could negatively affect parent-adolescent relationships (Nelson et al., 2017) and co-develop with worsened children’s mental health (Stone et al., 2016). Thus, studying the interactions between symptoms of parental burnout and parents’ mental health could be important in the area of neglect or violence toward children and in the context of positive dimensions of parent-adolescent relationships.

In the present study, we investigated three basic dimensions of parent-adolescent relationships: connectedness, involvement, and hostility (Nelson et al., 2017). Connectedness refers to emotional availability and responsivity, expressing love (both verbally and physically), affection and acceptance, feelings of closeness, warmth, and companionship (Burke et al., 2021). This dimension is strongly associated with adolescents’ mental and physical health (Viner et al., 2012). Parental involvement is characterized by the investment of time and resources into adolescents’ school and leisure activities (Burke et al., 2021). Particular forms of parental investment involve so-called shared activities that refer to occasions enjoyed by both members of the relationship (e.g., family mealtimes, family events, and shared leisure activities; Burke et al., 2021). Paternal hostility refers to criticism and rejection in parent-adolescent relationships (Burke et al., 2021). These parental behaviors are related to a higher risk of internalizing (e.g., depression) and externalizing (e.g., aggression) symptoms among adolescents (Putnick et al., 2015).

**The network analysis approach in investigating parental burden during the pandemic**

The network analysis approach was recently embraced in the field of psychopathology viewing the psychological constructs as dynamic systems of interacting elements (Borsboom & Cramer, 2013). This approach differs from the focus on unitary phenomena underlying psychological symptoms. Network analysis helps in determining which symptoms especially connect to others and could foster the activation of other psychopathological symptoms and maladaptive behaviors (Constantini et al., 2015).

Parental burnout can be conceptualized as a dynamic system of interacting variables, with three basic dimensions of parental burnout (overwhelming exhaustion related to the parental role, emotional distancing from one’s children, and a sense of ineffectiveness and of loss of accomplishment in the parental role; Mikolajczak & Roskam, 2018; Roskam, Raes, & Mikolajczak, 2017) interacting with each other and impacting the entire family (Blanchard & Heeren, 2020). Network analysis allows investigations (and clear visualizations) of the dynamic associations between many individual variables (such as parents’ and children’s personality or mental health), parental burnout, and various interpersonal processes depicted in family systems theories (Blanchard & Heeren, 2020). Studies using network analysis help to detect the central symptoms which can activate or cause other symptoms or processes within families with burnout parents (Cramer et al., 2010). For example, emotional distancing was an especially potent mechanism in activating other components of parental burnout, but also child neglect and violence (Blanchard et al., 2021). Thus, network analysis helps in detecting particular dimensions or symptoms of parental burnout which predict maladaptive parental behaviors (Kalkan et al., 2022). Network analysis also detects groupings of symptoms which helps in detecting naturally occurring groupings of symptoms and bridge symptoms between these communities of symptoms (Jones et al., 2021).

Since the network analysis approach is interested mostly in symptom-to-symptom associations, the integration of latent or aggregated variables into a network model is also possible (Bringemann & Erronen, 2018). Since a single indicator of a symptom could be associated with measurement error, using latent or aggregated measures of each node is accepted in the network analysis, or even beneficial (Cramer et al., 2010). Moreover, networks including aggregated indicators of nodes that are syndromes or clearly differentiated dimensions of a syndrome and its risk factors and consequences make it possible to model more complex associations between particular psychological and interpersonal processes. Recently, Schellekens et al. (2020) explored the interconnectedness of fatigue, depression, anxiety, potential risk, and protective factors in cancer patients, showing that ill acceptance among protective factors and helplessness among risk factors were the most central symptoms in the network. Thus, although the network analysis is conducted with nodes that are aggregated symptoms, the focus of the analysis is still on parts instead of wholes, which is characteristic of network analysis (Cramer et al., 2010). Similarly, in the case of parental burnout and its associations with its individual correlates and interpersonal consequences within the family, network analysis could be particularly helpful in detecting the central variables among the intrapersonal and interpersonal variables examined (Blanchard & Heeren, 2020).

Studies conducted in the context of the pandemic showed that parental burnout was strongly related to other indices of parental stress and mental health. Among parents, feeling worthless was a symptom that connected parental burnout with depression (Skjerdingstad et al., 2021). Parental depression and anxiety were higher among parents who experienced higher pandemic-related stress (Wu et al., 2020). Parents also reported experiencing chronic pandemic-related stress (Adams et al., 2021) that could result in pandemic burnout (Yıldırım & Solmaz, 2022). Moreover, studies indicated that parental stress, depression, and anxiety during the pandemic were related to worsened family functioning, including higher conflict and less closeness in relationships with children (Feinberg et al., 2022). Longitudinal studies conducted before the pandemic indicated that the directions of the associations between parental burden and relationships with children are to some extent mutual (Mikolajczak et al., 2019). Thus, worsened relations with children could affect exhaustion, feelings of ineffectiveness in the parental role, and emotional distancing from children.

The findings indicate that parental burnout during the pandemic, pandemic burnout, depression, and anxiety as well as relationships with children seem to constitute a particular network of mutual associations. The theoretical reasoning (e.g., the Family Stress Model; Masarik & Conger, 2017) also suggests that various stressors of internal and external nature with regard to family could impact parenting behaviors and result in child neglect or maltreatment. Thus, network approach could be particularly important to identify central symptoms and processes in this network that could be the targets of particular interventions or prevention strategies (Blanchard et al., 2021).

**The present study**

Our goal was to use network analysis to identify the most influential symptoms of parental burnout, pandemic stress, and parental mental health, and to investigate their associations with relationships with adolescent children. Contrarily to previous studies (Blanchard et al., 2021), we focused not only on the detrimental consequences of parental burnout for the relationship with children (parental neglect or violence) but on the basic dimensions of parent-adolescent relationships, namely: closeness, involvement, and hostility (Burke et al., 2021). First, we focused on investigating the central symptoms in the network of pandemic-related stress, parental burnout, parents’ internalizing symptomatology, and parent-child relationships that may maintain the network system (Constantini et al., 2015). Second, we investigated whether the studied symptoms constituted a unitary network or distinct communities (Jones et al., 2021). We wanted to identify the key processes that interacted with each other and mutually affected parental bonds with children. Thus, we also aimed to identify bridge symptoms responsible for links between the communities of symptoms studied in the network.

According to previous research (e.g., Skjerdingstad et al., 2021), we expected positive associations between dimensions of parental burnout and indicators of internalizing symptomatology among parents (Hypothesis 1). Moreover, we predicted that parental burnout and pandemic burnout would be positively associated (Hypothesis 2). The pandemic was a source of stress concerning health and job issues, but also inflicted an additional burden due to children’s remote learning and changes in daily routines of families, which could result in higher parental burnout (Prikhidko et al., 2020; Thomas et al., 2022). Previous studies indicated that parent who felt burned out showed less positive and more negative behaviors toward their children (Blanchard et al., 2021). Thus, in the present study we expected positive associations between dimensions of parental burnout and hostility toward the adolescent child (Hypothesis 3a), and negative associations between dimensions of parental burnout and shared activities and connectedness with the adolescent (Hypothesis 3b). In the context of worsening parenting among fathers and mothers who suffer from internalizing problems (Nelson et al., 2017), we predicted that parents’ levels of depression, anxiety, and pandemic burnout would be negatively associated with connectedness and shared activities with the adolescent (Hypothesis 4a), but positively associated with hostility toward the adolescent child (Hypothesis 4b).

We focused on adolescent children because of the typical high turbulence of t adolescence for the adolescents and their families (Keijsers et al., 2009), but also due to the risk revealed by the pandemic, namely reduced parental control and support associated with this period (Branje et al., 2002) that could constitute additional drawbacks for adolescents faced with consequences of the pandemic (e.g., remote learning, social isolation; Stavridou et al., 2020). Here, we applied the age range from 10 to 24 to more closely compare adolescent growth and popular understandings of this life phase (Sawyer et al., 2018).

**Method**

**Participants**

We used the following inclusion criteria for the study: (a) being a parent of at least one child; (b) age of at least one child should be between 10 and 24 years (Sawyer et al., 2018); (c) being able to speak Polish as we conducted the study in Polish. Four hundred and fifteen people responded to our invitation on various social media. Forty-one of them did not match the inclusion criteria. Six people indicated that they had no children, and thirty-five reported that their children were not between 10 and 24. The research sample consisted of three hundred and seventy-four parents (90.1% women) ranging from 40 to 61 (*M* = 42.9; *SD* = 5.8). The age of the youngest child in the family ranged from 4 months to 22 years (*M* = 12.4; *SD* = 4.1). The oldest child’s age ranged from 10 to 37 years (*M* = 17.1; *SD* = 4.2). The number of children ranged from one (27.81%) to six (0.3%). Among the participants, socioeconomic status was mainly average (75.4%) or higher (22.2%). Our main interest was not in sociodemographic variables; thus, we did not collect more precise data on the participants’ family status (e.g., single parenting) or job status.

The simulation and empirical studies indicated that adequate performance for estimating eight-node networks could be achieved using *N* = 50, but a correlation between true and obtained edges of *r* = 0.8 could be obtained from *N* = 250 (Epskamp & Fried, 2018). Our sample size was appropriate for estimating a network for 10 nodes with approximated 0.9 correlation with true edge according to the simulation procedure recommended by Epskamp and Fried (2018; see Figure S1 in Supplementary Materials).

**Measures**

***Parental Burnout Inventory*** (*PBI*; Roskam et al., 2018; Polish version: Szczygieł et al., 2020) consists of twenty-three items measuring exhaustion in the parental role (e.g., “I feel completely run down by my role as a parent”; nine items), contrast in parental self (e.g., “I’m no longer proud of myself as a parent”; six items), feelings of being fed up with parenting (e.g., “I can’t stand my role as father/mother any more”; five items), and emotional distancing from the children (e.g., “I do what I’m supposed to do for my child(ren), but nothing more”; three items). The items are rated on a 7-point Likert scale ranging from 0 (*never*) to 6 (*every day*). The scores were calculated as an average rating for the items. Higher scores indicated higher level of particular components of parental burnout. *PBI* has high internal consistency in Polish samples (Lin & Szczygieł, 2022; Roskam et al., 2021) and high convergent validity by its associations with indicators of parents’ poor mental health (e.g., depression, sleep disturbances, and burnout; Aunola, Sorkkila, & Tolvanen, 2020; Roskam et al., 2018; Szczygieł et al., 2020). In the present study, the reliability of each subscale of *PBI* was satisfactory (see Table 1).

***Pandemic burnout*** was assessed with the COVID-19-Burnout Scale (*COVID-19-BS*; Yıldırım & Solmaz, 2022; Polish version: [hidden for blind review]). *COVID-19-BS* consists of ten items (e.g., “When you think about COVID-19 overall, how often do you feel disappointed with people?”). Each item is rated on a 5-point Likert scale ranging from 1 (*never*) to 5 (*always*). The scores were calculated as an average rating for the items. Higher scores indicated higher pandemic burnout. Previous studies indicated a positive association between *COVID-19-BS* scores and depression ([hidden for blind review]). *COVID-19-BS* was validated by its positive associations with depressive symptoms (Yıldırım & Solmaz, 2022), intolerance of uncertainty (Haktanir et al., 2022), and COVID stress (Yıldırım et al., 2021). The reliability of *COVID-19-BS* exceeded α = 0.90 in the majority of the previous studies (Hatkanir et al., 2022; [hidden for blind review]). The reliability of *COVID-19-BS* in the current study was also satisfactory (Table 1).

***The Hospital Anxiety Depression Scale*** (*HADS*; Zigmond & Snaith, 1983; Polish version: Nezlek et al., 2021) consists of fourteen items measuring depression (e.g., “I feel as if I am slowed down”; seven items) and anxiety (e.g., “I feel tense or wound up”; seven items). Each item is rated on a four-point scale with answers describing the intensity of symptoms ranging from 0 (e.g., “Not at all”) to 3 (e.g., “Most of the time”). The scores of depression and anxiety scales were calculated as an average of the items measuring the particular construct. Higher scores indicated higher depression and anxiety. Previous studies using *HADS* in Polish samples indicated stability of its internal structure (Nezlek et al., 2021) and validity (Watrowski & Rohde, 2014). In the present study, both depression and anxiety scales had satisfying reliability (Table 1).

***The Parent-Adolescent Relationship Scale*** – Parent report version (*PARS*; Burke et al., 2021). *PARS* consists of 15 items measuring: connectedness (e.g., “I encourage my teenager to get support from me or others”; six items), shared activities (e.g., “I play sport or do other physical activities with my teenager”; four items), and parental hostility (e.g., “I make negative comments about my teenager to others”; five items). Parents rated each item on a 6-point Likert scale ranging from 0 (*not at all true*) to 5 (*nearly always or always true*). Scores were calculated as average ratings for the items. Higher scores indicated higher shared activities, connectedness, and hostility toward the child. The Polish version of *PARS* was developed by a back-translation procedure conducted by two psychologists and one professional English editor. The reliability of subscales of *PARS* was satisfactory in previous studies (e.g., Dittman et al., 2022) and in the present study (Table 1). The validity of *PARS* was verified in previous studies by correlations with positive (e.g., support) and negative (e.g., inconsistent discipline) dimensions of parenting, and indicators of positive adolescents’ functioning as well as their emotional or behavioral problems (e.g., emotional difficulties; Burke et al., 2021; Dittman et al., 2022).

**Procedure**

The study was approved by the institutional review board [hidden for blind review]. We contacted the participants via social media and invited them to participate as volunteers (without remuneration) in an on-line survey. The participants were informed about the aim of the study and were asked for their informed consent before participation. Afterward, they filled out a set of questionnaires consisting of the measures described above and a short sociodemographic survey (e.g., age, number of children, age of the children, socioeconomic status). After the survey, the participants were thanked, debriefed, and had an opportunity to contact the first author via e-mail in case of questions or concerns. We collected the data from September 2021 to February 2022.

**Network analysis**

We conducted our analysis following the recent guidelines for applying network analysis in clinical psychology (Epskamp & Fried, 2018) and previous network analysis concerning parental burnout (Blanchard et al., 2021). In the beginning, we prepared data by applying the nonparanormal transformation using the R package *huge* (Jiang et al., 2019). This transformation helps to obtain multivariate normal distribution of data required for network analysis (Epskamp et al., 2018). Next, we screened for redundant symptoms (referred to in the network approach as ‘nodes’). We checked if the correlation matrix was positive definite, which reflects that nodes were not a linear combination of each other. We also implemented the Hittner method for comparing dependent correlations (Hittner, et al., 2003) to detect pairs of nodes that correlated strongly with each other (*r* > .50) and correlated similarly with more than 75% of other nodes in the network. We used the goldbricker function of the R package *networktools* (Jones, 2018).

In the next step, we used a graphical Gaussian model (*GGM*) to estimate the undirected network. Edges between nodes in this network indicate independent relationships between each node controlling for the effect of all other nodes in the network (Epskamp & Fried, 2018). We used the graphical *LASSO* (Least Absolute Shrinkage and Selection Operator) algorithm to regularize our network. First, this algorithm calculated partial correlations between nodes and then shrank trivially small correlations to zero (Epskamp et al., 2018). To select the best model of the network, we applied the Extended Bayesian Information Criterion (*EBIC*) with hyperparameter gamma (γ) set to 0.5 as recommended to achieve higher confidence that our edges were genuine (Epskamp et al., 2018). We used the R packages *ggraph* and *bootnet* to select the best network and to estimate the stability of edges using confidence regions for edge weights (Epskamp et al., 2018). We assessed the centrality of nodes in the network using Expected Influence (*EI*), which is the sum of the edge weights incident on a given node, including positive and negative values (Robinaugh et al., 2016). To investigate the stability of centrality indices, we used the correlation stability coefficient (*CS*-coefficient) which “represents the maximum proportion of cases that can be dropped, such that with 95 % probability the correlation between original centrality indices and centrality of networks based on subsets is 0.7 or higher” (Epskamp et al., 2018, p. 200) and should be above 0.5 (Epskamp et al., 2018). We also implemented tests of differences between expected influences of nodes to detect symptoms that were central and stronger than other nodes (Epskamp et al., 2018).

We investigated communities of symptoms (nodes) using the spin glass algorithm, which is a modularity-based community detection procedure suitable for uncovering the structure of relatively small networks with negative edge values (Blanchard et al., 2021). We used the *spinglass.community* function (γ = 1, start temperature = 1, stop temperature = .01, cooling factor = .99, spins = 7) of the R package *igraph.* Finally, we tried to identify nodes that were bridges between communities. We computed the Bridge Expected Influence (1-step) using the *bridge* function of the R package *networktools* (Jones, 2018). Bridge expected influence (1-step) reflects the sum of the edge weights connecting a given node to all nodes in the other community or communities (Jones et al., 2019). We also inspected Bridge Expected Influence (2-step) that also indicates the indirect effect that a particular node may have on other communities through other nodes (e.g., an indirect effect on node C as in A → B → C; Jones, 2018).

**Results**

**Descriptive statistics**

Means, standard deviation, skewness, kurtosis, and correlations with sociodemographic variables regarding the studied variables (before nonparanormal transformation) are given in Table 1.

*Table 1*

Descriptive statistics of studied variables

| Variable | *M* | *SD* | Cronbach’s α | Skewness | Kurtosis | Age | Gender | Number of children | Youngest child | Socioeconomic status |
| --- | --- | --- | --- | --- | --- | --- | --- | --- | --- | --- |
| 1. Pandemic burnout | 2.60 | 0.89 | .91 | 0.17 | –0.58 | –.09 | .17** | –.12* | .02 | .01 |
| **Parental burnout** | | | | | | |  |  |  |  |
| 2. Exhaustion in the parental role | 1.17 | 1.33 | .95 | 1.57 | 1.89 | –.04 | .06 | .04 | –.16** | –.01 |
| 3. Contrast in parental self | 1.08 | 1.36 | .94 | 1.67 | 2.26 | –.02 | .05 | .05 | –.08 | –.02 |
| 4. Feelings of being fed up with parenting | 0.99 | 1.29 | .93 | 1.63 | 1.93 | –0.02 | .07 | .03 | –.20* | .01 |
| 5. Emotional Distancing from children | 0.85 | 1.17 | .82 | 1.85 | 3.45 | .03 | .01 | –.01 | .01 | .02 |
| **Parent-Adolescent Relationships** | | | | | | |  |  |  |  |
| 6. Shared Activities | 3.35 | .99 | .73 | –0.71 | 0.48 | –.08 | .01 | –.06 | –.05 | .06 |
| 7. Connectedness | 4.41 | 0.73 | .85 | –2.00 | 5.99 | –.02 | .18*** | .04 | –.04 | .01 |
| 8. Hostility | 1.85 | 1.00 | .78 | 0.38 | –0.26 | .01 | .10 | .02 | –.03 | –.03 |
| **Parent’s mental health** | | | | | | |  |  |  |  |
| 9. Anxiety | 1.04 | 0.59 | .88 | 0.64 | 0.15 | –.06 | .16** | –.12* | -.05 | –.09 |
| 10. Depression | 0.66 | 0.51 | .81 | 0.69 | –0.06 | .01 | .07 | –.10* | .02 | –.07 |

* *p* < .05, ** *p* < .01, *** *p* < .001

The Hittner procedure indicated that emotional exhaustion and feeling of being fed up were redundant (*r* = .94; *p* < .001). Thus, we calculated the mean score for these variables and named it *Exhaustion and feelings of being fed up.* After the nonparanormal transformation of scores, we calculated the correlations presented in Figure 1.


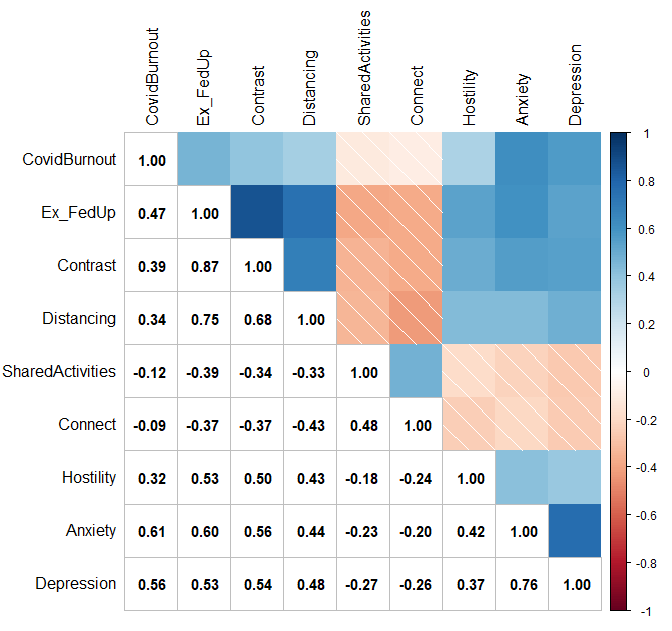


Figure 1

*Pearson product-moment correlations between each nonparanormal-transformed variable*

**Graphical *LASSO* network**

We present the graphical *LASSO* in Figure 2. Edges represent partial correlations between nodes. Among parental burnout symptoms, the largest edge weight was between emotional exhaustion/feelings of being fed up and a sense of ineffectiveness in the parental role (*r* = .64) and between exhaustion and emotional distancing from the children (*r* = .37). Depression correlated strongly with anxiety (*r* = .55). Pandemic burnout correlated with depression (*r* = .17) and anxiety (*r* = .29). Exhaustion correlated with higher parental hostility (*r* = .15), fewer shared activities (*r* = –.12), and higher parental anxiety (*r* = .15). Emotional distancing was negatively associated with connectedness (*r* = –.19). To estimate the precision of the edge weights, we bootstrapped confidence intervals for each of the edge weights (n = 1000 boots; Epskamp et al., 2018) and we performed bootstrapped difference tests (α =.05). The results show that the edges are stable (*CS* [cor = .70] = .75), and that the strongest and the weakest edges are significantly different from one another (Supplementary Material).


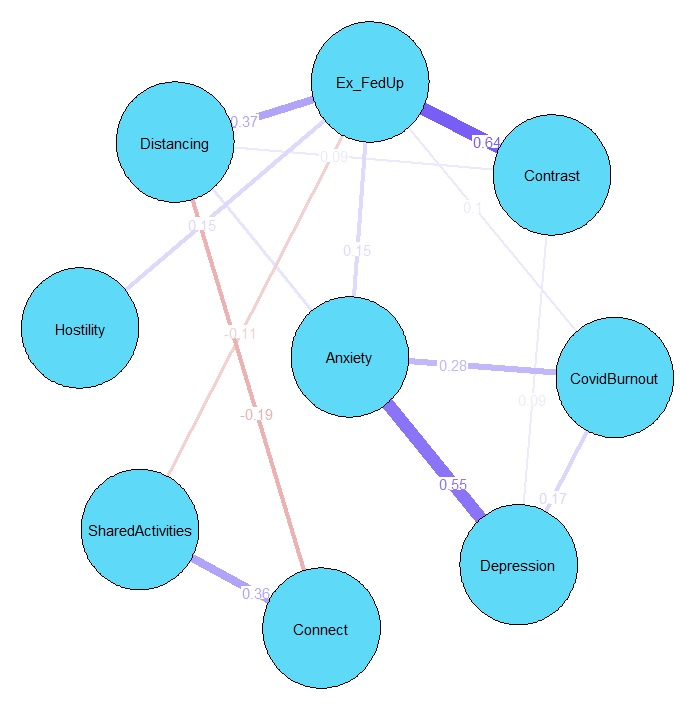


Fig. 2. Graphical Gaussian Model Network Constructed via the Graphical *LASSO*.

*Note*. Blue lines represent positive regularized partial correlations. Red lines represent negative regularized partial correlations. The numbers represent regularized partial correlations. Ex_FedUp = Emotional exhaustion and feelings of being fed up with parenting; Contrast = Contrast in parental self.

We report the expected influence (*EI*) values in Figure 3. Emotional exhaustion and feelings of being fed up with parenting (*EI* = 1.29), anxiety (*EI* = 0.98), and depression (*EI* = 0.91) had the highest expected influence values. To ensure stability of these centrality estimates, we performed a person-dropping bootstrap procedure (Epskamp et al., 2018) and we confirmed high stability of these expected influence values (see Fig. S4 in Supplementary Materials). Stability of expected influence was appropriate (*CS* [cor = .70] = .75). We also performed a bootstrapped difference test which revealed that the most central nodes (i.e., emotional exhaustion/feelings of being fed up and anxiety) have significantly higher expected influence estimates than less central nodes (see Fig. S5 in Supplementary Materials).


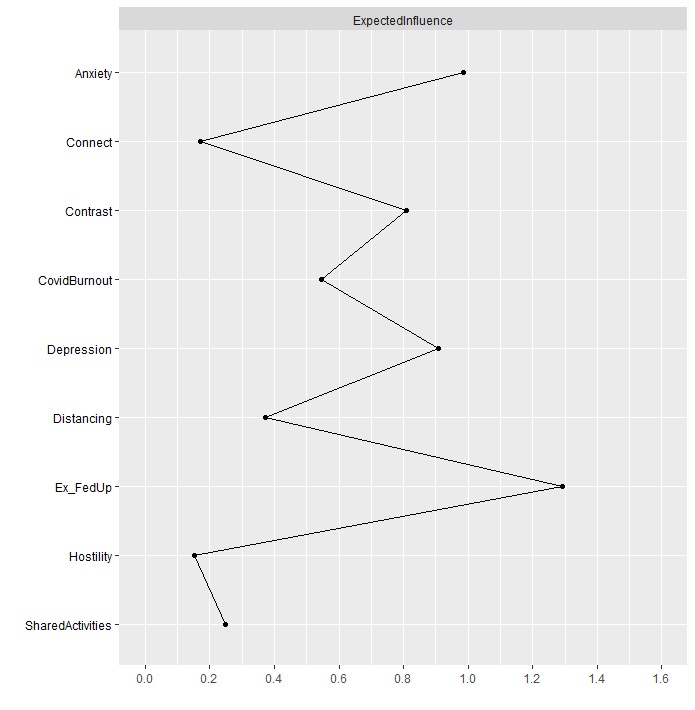


Fig. 3. Expected Influence Estimates of the Graphical *LASSO*.

*Note*. Ex_FedUp = Emotional exhaustion and feelings of being fed up with parenting; Contrast = Contrast in parental self.

**Communities of nodes and bridge nodes**

The spin glass algorithm detected four communities of nodes. The first community included emotional distance, exhaustion, and ineffectiveness in the parental role, which could be regarded as manifestations of parental burnout. The second community consisted of shared activities and connectedness. The third community included hostility, and the fourth community consisted of anxiety, depression, and pandemic burnout. Fig. 4 shows bridge expected influence (*BEI*) values for all nodes, revealing that exhaustion/feelings of being fed up (*BEI* = .29), depression (*BEI* = .20), anxiety (*BEI* = .15), and parental hostility (*BEI* = .15) had high bridge expected influence value. These symptoms positively activated other symptoms in the network. Contrarily, connectedness (*BEI* = –.19) could be a deactivator of other symptoms in the network. We performed a person-dropping bootstrap, which indicated that bridge expected influence values were stable (see Fig. S3 in Supplementary materials). The stability of bridge expected influence was appropriate (*CS* [cor = .70] = .75). A bootstrapped difference test showed that parental hostility, emotional exhaustion, anxiety, and pandemic burnout had significantly higher bridge expected influence than the remaining variables (Fig. S6 in Supplementary Materials). Moreover, bridge expected influence (2-step) analysis showed that emotional exhaustion/feelings of being fed up with parenting (*BEI* [2-step] = .44) and anxiety (*BEI* [2-step] = .45) remained the most influential nodes. However, depression (*BEI* [2-step] = .39), contrast in parental self (*BEI* [2-step] = .32), parental hostility (*BEI* [2-step] = .33), and pandemic burnout (*BEI* [2-step] = .27) could indirectly affect other nodes. Contrarily, connectedness (*BEI* [2-step] = –.35) and shared activities (*BEI* [2-step] = –.35) could be nodes that deactivate other nodes in the network both directly and indirectly.

**A.**


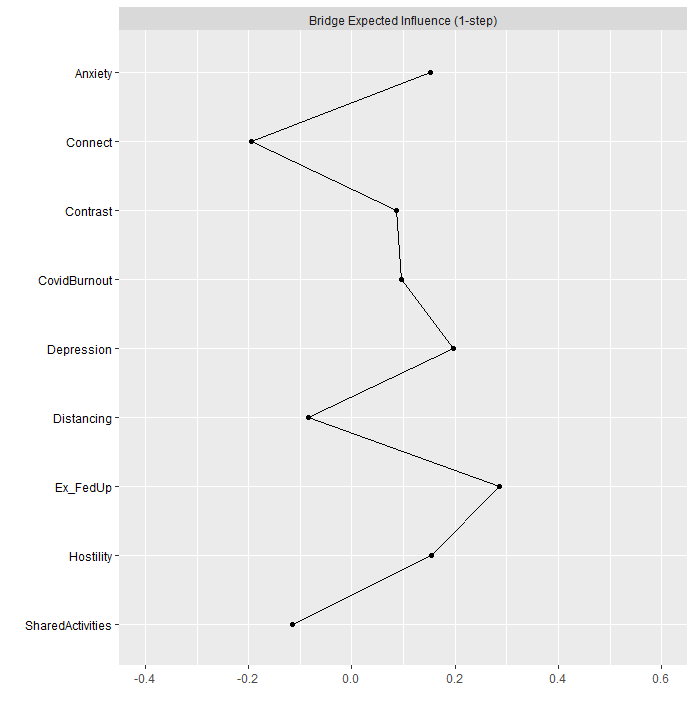


**B.**


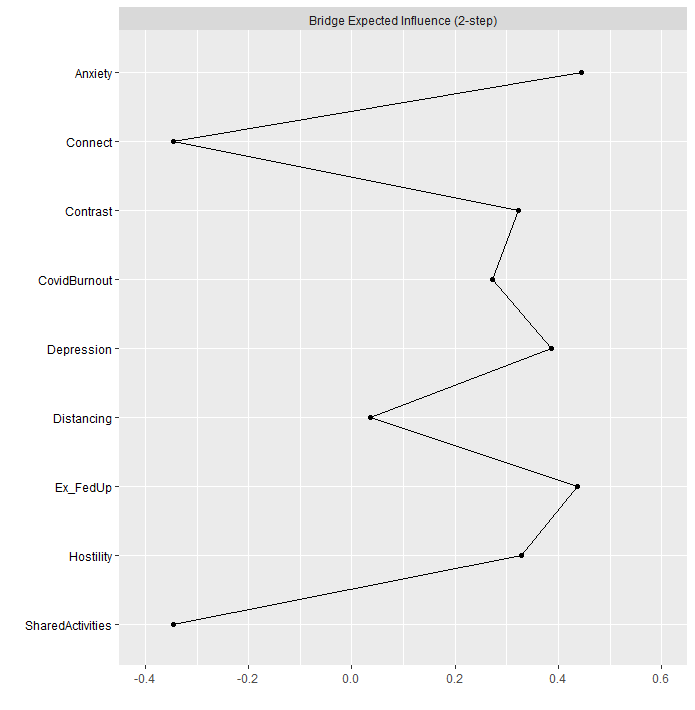


Fig. 4. Bridge Expected Influence (1 -step – panel A; 2-step – panel B) Estimates of the Graphical *LASSO*.

*Note*. Ex_FedUp = Emotional exhaustion and feelings of being fed up with parenting; Contrast = Contrast in parental self.

Bridge symptoms may also be estimated for particular communities of nodes. Thus, we estimated bridge expected influence for communities of parental burnout and parent-adolescent relationships, communities of pandemic burnout/parental internalizing symptoms (depression and anxiety) and parent-adolescent relationships as well as communities of parental and pandemic burnout/parental internalizing symptoms (Table S1 in Supplementary Materials). The analysis indicated that hostility had the highest bridge expected influence (*BEI* = .15) while distancing (*BEI* = –.19) and connectedness (*BEI* = –.19) had negative bridge expected influence. Thus, parental hostility could activate parental burnout, while connectedness as a dimension of parent-adolescent relationships could deactivate parental burnout, and distancing from the children could deactivate positive parent-adolescent relationships. There were no bridge symptoms between pandemic burnout/parental internalizing symptoms and parent-adolescent relationships. Emotional exhaustion and feelings of being fed up (*BEI* = .25) and depression (*BEI* = .20) were bridge symptoms between parental and pandemic burnout.

**Discussion**

Parental burden during the pandemic consisted of difficulties caused by the pandemic and preventive measures applied by governments to stop the spread of the virus, which strongly affected parenting and daily routines in families (Feinberg et al., 2022). Moreover, these difficulties caused severe stress which affected family relationships and parenting (Masarik & Conger, 2017). Studies demonstrated increased parental burnout during the pandemic (van Bakel et al., 2022), increased internalizing problems (Wu et al., 2020) and chronic pandemic-related stress among parents (Adams et al., 2021) that could lead to pandemic burnout (Yıldırım & Solmaz, 2022). These parental mental health conditions affected parents’ relationships with children (Feingold et al., 2022). Moreover, parental relationships with adolescents could be at particular risk (Donker et al., 2021) due to the typically high turbulence characterizing adolescence as a developmental period. Given the mostly mutual nature of the relationships between parental burnout, parental internalizing symptoms, and parent-child relationships (Mikolajczak et al., 2019), network analysis was especially useful for studying associations between parental burnout and parent-child relationships (Blanchard et al., 2021). Thus, the current study investigated associations between parental and pandemic burnout, parental internalizing symptomatology, and parent-adolescent relationships using a network analysis approach.

Correlation analysis supported the hypotheses on the positive associations between dimensions of parental burnout, internalizing symptomatology (e.g., anxiety, depression) among parents (Hypothesis 1), and pandemic burnout (Hypothesis 2). Parents who reported higher burnout also reported being more anxious and depressed during the previous two weeks and being more burned out by the pandemic. Correlation analysis also supported Hypotheses 3a and 3b, in which we expected positive associations between parental burnout and hostility toward the children and negative associations between parental burnout and shared activities and connectedness with the children. Hypothesis 4a was partially confirmed, as depression and anxiety reported by parents, but not pandemic burnout, correlated negatively with shared activities and connectedness with the adolescents. Among the parents, anxiety, depression, and pandemic burnout were positively associated with hostility toward the adolescent children, which confirmed Hypothesis 4b. However, network analysis showed that these direct associations were quantified by the centrality of particular symptoms in communities of symptoms of parental burnout, parents’ mental health, and parent-adolescent relationships.

The graphical Gaussian model indicated that emotional exhaustion and feelings of being fed up with parenting were central symptoms of parental burnout, and generally central symptoms in the network. Components of parental burnout, namely emotional exhaustion, emotional distancing, feelings of being fed up with parenting, and contrast in parental self constituted a community. Our results are in concordance with previous findings about the central role of emotional exhaustion in psychological burnout (Halbesleben & Bowler, 2007). Moreover, our study showed that emotional exhaustion had the highest mean item score. Therefore, emotional exhaustion as a symptom of parental burnout is not only influential, but also prevalent. In previous studies on parental burnout that used network analysis, emotional distancing appeared as a central symptom in the network of parental burnout (Blanchard et al., 2021), although in the latter study, contrast in parental self formed its own community. Longitudinal analyses indicated emotional exhaustion as a key symptom in parental burnout (Roskam & Mikolajczak, 2021). We also showed that emotional exhaustion and feelings of being fed up with parenting were strongly correlated and appeared redundant according to the Hittner dependent correlations method (Hittner et al., 2003). Thus, our study indicated that during the pandemic parents reported emotional exhaustion as the central symptom of parental burnout. Emotional exhaustion appears in the first phase of the course of parental burnout (Roskam & Mikolajczak, 2021). Thus, the centrality of this symptom could also indicate that many parents experienced a heightened emotional burden of parenting during the pandemic.

Internalizing symptoms were prevalent during the pandemic, with higher frequency of anxiety symptoms (Johnson et al., 2021). Our results indicated that anxiety was positioned second in terms of centrality in the network of parental burnout, pandemic burnout, and parent-adolescent relationships. Although the regularized partial correlations showed that anxiety was related mainly to emotional exhaustion, its indirect associations with worsened connectedness with the adolescents and emotional distancing from the children were documented by high bridge expected influence (2-step). Feelings of tension, inability to relax, worrying, and sudden feelings of panic that constitute anxiety (Zigmond & Snaith, 1983) resulting from the pandemic and all its consequences (Arora et al., 2022) could activate more tension in parenting. In turn, this tension could result in worsened parent-adolescent relationships.

Parents who experienced strong emotional exhaustion and distancing expressed fewer shared activities and less connectedness with their adolescent children. This result showed that increased parental burnout could result not only in the risk of child maltreatment (Mikolajczak et al., 2018) but also in worsening of positive parenting behaviors (Belson et al., 2017). Moreover, emotional exhaustion was positively associated with parental hostility. This result is in line with previous findings concerning a positive association between parental burnout and hostility (Chen et al., 2022; Knox et al., 2011). Thus, parental emotional exhaustion seems to be the central symptom of parental burnout, which is associated with decreased positive parenting and increased hostility toward the adolescent children. Similarly to previous findings (Donker et al., 2021), our analysis also showed that it was not so much the pandemic stress *per se*, but rather increased internalizing symptoms and parental burnout that could affect the relationships between parents and adolescents.

Subcommunities within the network, detected by the Spinglass algorithm, showed that parental burnout symptoms formed a syndrome which was independent from depression, anxiety, and pandemic burnout that formed another subcommunity referring to parents’ mental health. Moreover, parental burnout was independent from two communities consisting of parental behaviors (e.g., shared activities and connectedness, and hostility). This result replicated our measurement model. However, in a previous network analysis of parental burnout and parenting behaviors (Blanchard et al., 2021), two dimensions of parental burnout (e.g., emotional distance and emotional exhaustion) formed a subcommunity of symptoms with neglect and violence toward children. Recent network analysis of parental burnout confirmed that symptoms of parental burnout, parental neglect, and violence are independent syndromes of symptoms (Kalkan et al., 2022). Also the present results confirmed the independence of symptoms of parental burnout from the symptoms of parental neglect. Moreover, the present study was the first to use network analysis to show the independence of parents’ internalizing problems (e.g., depression, anxiety, and pandemic burnout) from the parental burnout symptoms. This finding is in line with previous results on factorial distinctiveness between parental burnout and depression (Mikolajczak et al., 2020). However, since the present analysis was conducted on nodes which were aggregated indicators of the particular syndrome of symptoms (e.g., dimensions of parental burnout, depression, and anxiety), future studies should investigate the communities in the network of parental burnout and parents’ internalizing symptoms at the level of the symptoms of these constructs. Such analysis could better indicate the dynamic associations between symptoms, better detect the groupings of symptoms, and help in identifying significant bridge symptoms between these communities of symptoms (see Jones et al., 2021).

Our study has some limitations. Firstly, cross-sectional data precludes causal interpretations. Since a network approach enables examination of dynamic associations between symptoms, the cross-sectional design of the study gives a limited picture of the dynamics of the associations between symptoms of parental burnout, parents’ internalizing symptoms and their relationships with children. Longitudinal research should support the bridge centrality of emotional exhaustion and anxiety in the network. Moreover, experience sampling methods may be used to analyze the dynamics in the network including variables that reflect reactions to short-term stressors or persistent stressors (such as the pandemic) and parental burnout (cf., Bringmann et al., 2013). Although the present study showed the central role of emotional exhaustion in parental burnout only in a cross-sectionally measured network of symptoms, other studies also indicated that in temporal networks, emotional exhaustion plays a central role and activates other symptoms of parental burnout (Blanchard, Hoebeke, & Heeren, 2022).

Secondly, generalizability of our results may be limited due to the possibility of self-selection bias in the recruitment procedure. Parents who felt burned out could be reluctant to participate in the study due to lack of compensation or in order to avoid the difficult topic the study addressed. However, when using cut-off points for the *PBA* (86 and 92), the number of parents reporting significant levels of parental burnout were 6.8% and 4.6% which was comparable to previous studies conducted in large samples of parents in Poland (4.1% and 3.2% ; Szczygieł et al., 2021). Since the main goal of our study was to examine associations between parental burnout and parent-adolescent relationships, the mean age of the parents in our sample was higher compared to previous studies conducted among Polish parents (Roskam et al., 2021; Szczygieł et al. 2021). Thus, the present study could examine the associations between parental burnout and parent-child relationships for later stages of family development compared to previous studies, mostly conducted among parents of young children.

The third limitation is the high proportion of women in the sample. Although the prevalence of parental burnout is higher among mothers, the consequences of parental burnout were more detrimental for fathers (e.g., neglectful behaviors toward children were more common in burned-out fathers than in burned-out mothers; Roskam & Mikolajczak, 2020). Thus, the lack of balance between mothers and fathers in our study could result in an overestimation of parental burnout, but in an underestimation of its examined associations. Moreover, fathers were more vulnerable to the imbalance of risks over resources, which is a core characteristic of parental burnout (Roskam & Mikolajczak, 2020). Thus, the low number of fathers in our study could result in an underestimation of the associations between parental burnout and parental mental health indicators. It should also be mentioned that unique norms, expectations, and socialization practices surrounding gender and parenting could affect the imbalance of fathers and mothers in the sample. The image of an ideal mother and an ideal father in the cultural area that includes Poland is substantially similar (Lin et al., 2023), but in Polish culture, demands concerning motherhood are stronger than those concerning fatherhood (Wołowicz-Ruszkowska, 2016). Thus, the imbalance of mothers and fathers could result in examining mostly the costs of parenting for the more culturally-burdened parent group. It could also be significant in the context of the pandemic, which caused more burden to mothers (Thomas et al., 2022). Therefore, future studies should focus on ensuring a balanced proportion of fathers and mothers among the participants. However, our results concerning the central role of emotional exhaustion in parental burnout and in parent-adolescent relationships during the pandemic and the lack of direct association with pandemic-related stress converged with results in large samples (Donker et al., 2021).

Finally, we failed to control for sociodemographic variables such as employment status and family type (Mikolajczak et al., 2021). However, introducing sociodemographic factors into the network makes it more difficult to estimate its parameters and understand the associations obtained. Nevertheless, future studies should control for sociodemographic variables.

**Conclusion**

Emotional exhaustion and feelings of being fed up with parenting were the central symptoms in the network of parental burnout, pandemic burnout, parental internalizing symptoms, and parent-adolescent relationships. Exhausted parents were emotionally distant, involved in fewer shared activities with their adolescent children, and reported less connectedness but higher hostility. Moreover, pandemic stress was associated with higher emotional exhaustion in parents through heightened anxiety (Masarik & Conger, 2017). These results indicate that interventions directed to parents should address primarily emotional exhaustion and anxiety. Such interventions could be conducted in small groups focused on education about the symptoms of burnout and intended to help to develop better strategies to cope with parental and external stress (Lindström et al., 2016). Mental health professionals, teachers, and social workers should recognize anxiety as a risk factor of heightened emotional exhaustion in the parental role to reflect with their clients on these dimensions of their functioning in clinical settings (Marchetti et al., 2020).

**References**

Adams, E.L., Smith, D., Caccavale, L.J., & Bean, M.K. (2021). Parents are stressed! Patterns of parent stress across COVID-19. *Frontiers in Psychiatry*, *12*, 626456. https://doi.org/10.3389/fpsyt.2021.626456

Aguiar, J., Matias, M., Braz, A.C., César, F., Coimbra, S., Gaspar, M.F., & Fontaine, A.M. (2021). Parental burnout and the COVID-19 pandemic: How Portuguese parents experienced lockdown measures. *Family Relations*, *70*, 927–938. https://doi.org/10.1111/fare.12558

Arora, T., Grey, I., Östlundh, L., Lam, K., Omar, O.M., & Arnone, D. (2022). The prevalence of psychological consequences of COVID-19: A systematic review and meta-analysis of observational studies. *Journal of Health Psychology*, *27*, 805–824. https://doi.org/10.1177/1359105320966639

Aunola, K., Sorkkila, M., & Tolvanen, A. (2020). Validity of the Finnish version of the Parental Burnout Assessment (PBA). *Scandinavian Journal of Psychology, 61*, 714–722. https://doi.org/10.1111/sjop.12654

Blanchard, M.A., & Heeren, A. (2020). Why we should move from reductionism and embrace a network approach to parental burnout. New Directions for Child and Adolescent Development, 174, 159–168. https://doi.org/10.1002/cad.20377

Blanchard, M.A., Hoebeke, Y., & Heeren, A. (2022, March 28). Parental burnout features and the family context: A temporal network approach in mothers. https://doi.org/10.31234/osf.io/aef27

Blanchard, M.A., Roskam, I., Mikolajczak, M., & Heeren, A. (2021). A network approach to parental burnout. *Child Abuse & Neglect*, *111*, 104826. https://doi.org/10.1016/j.chiabu.2020.104826

Borsboom, D., & Cramer, A.O. (2013). Network analysis: an integrative approach to the structure of psychopathology. *Annual Review of Clinical Psychology*, *9*, 91–121. https://doi.org/10.1146/annurev-clinpsy-050212-185608

Branje, S., Laursen, B., & Collins, W.A. (2012). Parent-child communication during adolescence. In A.L. Vangelisti (Ed.), *Routledge Handbook of Family Communication* (2nd ed., pp. 271–286). Routledge.

Branje, S.J., van Aken, M.A.G., & van Lieshout, C.F.M. (2002). Relational support in families with adolescents. *Journal of Family Psychology*, 16, 351–362. https://doi.org/10.1037/0893-3200.16.3.351

Bringmann, L. F., & Eronen, M. I. (2018). Don't blame the model: Reconsidering the network approach to psychopathology. *Psychological Review, 125*, 606–615. https://doi.org/10.1037/rev0000108

Bringmann, L. F., Vissers, N., Wichers, M., Geschwind, N., Kuppens, P., Peeters, F., Borsboom, D., & Tuerlinckx, F. (2013). A network approach to psychopathology: New insights into clinical longitudinal data. *PLoS ONE, 8,* Article e60188. https://doi.org/10.1371/journal.pone.006018

Burke, K., Dittman, C.K., Haslam, D., & Ralph, A. (2021). Assessing critical dimensions of the parent-adolescent relationship from multiple perspectives: Development and validation of the Parent-Adolescent Relationship Scale (PARS). *Psychological Assessment*, *33*, 395–410. https://doi.org/10.1037/pas0000992

Chen, B. B., Qu, Y., Yang, B., & Chen, X. (2022). Chinese mothers' parental burnout and adolescents' internalizing and externalizing problems: The mediating role of maternal hostility. *Developmental Psychology, 58*, 768–777. https://doi.org/10.1037/dev0001311

Costantini, G., Epskamp, S., Borsboom, D., Perugini, M., Mõttus, R., Waldorp, L.J., & Cramer, A.O.J. (2015). State of the aRt personality research: A tutorial on network analysis of personality data in R. Journal of Research in Personality, 54, 13–29. https://doi.org/10.1016/j.jrp.2014.07.003

Cramer, A., Waldorp, L., Van der Maas, H., & Borsboom, D. (2010). Comorbidity: A network perspective. *Behavioral and Brain Sciences, 33*, 137–150. doi:10.1017/S0140525X09991567

De Goede, I.H.A., Branje, S.J.T., & Meeus, W.H.J. (2009). Developmental changes in adolescents’ perceptions of relationships with their parents. *Journal of Youth and Adolescence*, 38, 75–88. https://doi .org/10.1007/s10964-008-9286-7

Dittman, C. K., Burke, K., Haslam, D., & Ralph, A. (2022). Assessing adolescent functioning from different perspectives: Extending the validation of the Adolescent Functioning Scale (AFS). *Child Psychiatry and Human Development*. Advance online publication. https://doi.org/10.1007/s10578-022-01428-2

Donker, M.H., Mastrotheodoros, S., & Branje, S. (2021). Development of parent-adolescent relationships during the COVID-19 pandemic: The role of stress and coping. Developmental Psychology, 57, 1611–1622. https://doi.org/10.1037/dev0001212

Epskamp, S., & Fried, E.I. (2018). A tutorial on regularized partial correlation networks. Psychological Methods, 23, 617–634. https://doi.org/10.1037/met0000167

Epskamp, S., Borsboom, D. & Fried, E.I. (2018) Estimating psychological networks and their accuracy: A tutorial paper. *Behavior Research Methods*, *50***,** 195–212. https://doi.org/10.3758/s13428-017-0862-1

Feinberg, M.E.A., Mogle, J., Lee, J.K., Tornello, S.L., Hostetler, M.L., Cifelli, J.A., Bai, S., & Hotez, E. (2022). Impact of the COVID-19 pandemic on parent, child, and family functioning. *Family Process*, *61*, 361–374. https://doi.org/10.1111/famp.12649

Finkenauer, C., Willems, Y.E., Weise, M., & Bartels, M. (2019). The social context of adolescent relationships. In T. Burns & F. Gottschalk (Eds.), *Educating 21st century children: Emotional well-being in the digital age*. OECD Publishing, Paris, https://doi.org/10.1787/f71c8860-en

Haktanir, A., Can, N., Seki, T., Kurnaz, M. F., & Dilmaç, B. (2022). Do we experience pandemic fatigue? current state, predictors, and prevention. *Current Psychology, 41*, 7314–7325. https://doi.org/10.1007/s12144-021-02397-w

Halbesleben, J.R.B., & Bowler, W.M. (2007). Emotional exhaustion and job performance: The mediating role of motivation. Journal of Applied Psychology, 92, 93–106. https://doi.org/10.1037/0021-9010.92.1.93

Hittner, J.B., May, K., & Silver, N.C. (2003). A Monte Carlo evaluation of tests for comparing dependent correlations. *The Journal of General Psychology, 130*,149–168. https://doi.org/10.1080/00221300309601282.

Johnson, M.S., Skjerdingstad, N., Ebrahimi, O.V., Hoffart, A., & Johnson, S.U. (2021a). Parenting in a pandemic: Parental stress, anxiety and depression among parents during the government-initiated physical distancing measures following the first wave of COVID-19. *Stress and Health.* Advance online publication. https://doi.org/10.1002/smi.3120

Johnson, M. S., Skjerdingstad, N., Ebrahimi, O. V., Hoffart, A., & Urnes Johnson, S. (2021b). Mechanisms of parental distress during and after the first COVID-19 lockdown phase: A two-wave longitudinal study. *PloS one, 16*(6), Article e0253087. https://doi.org/10.1371/journal.pone.0253087

Jones, P.J. (2018). *Networktools: Tools for identifying important nodes in networks* (R Package Version 1.1.1.).

Jones, P.J., Ma, R., & McNally, R.J. (2021). Bridge centrality: A network approach to understanding comorbidity. *Multivariate Behavioral Research*, *56*, 353–367. https://doi.org/10.1080/00273171.2019.1614898

Kalkan, R.B., Blanchard, M.A., Mikolajczak, M., Roskam, I, & Heeren, A. (2022). Emotional exhaustion and feeling fed up as the driving forces of parental burnout and its consequences on children: insights from a network approach. *Current Psychology*. Advance online publication. https://doi.org/10.1007/s12144-022-03311-8

Keijsers, L., Frijns, T., Branje, S.J.T., & Meeus, W. (2009). Developmental links of adolescent disclosure, parental solicitation, and control with delinquency: Moderation by parental support. *Developmental Psychology*, *45*, 1314–1327. https://doi.org/10.1037/a0016693

Kerr, M.L., Fanning, K.A., Huynh, T., Botto, I., & Kim, C.N. (2021). Parents’ self-reported psychological impacts of COVID-19: Associations with parental burnout, child behavior, and income. Journal of Pediatric Psychology, 46, 1162–1171. https://doi.org/10.1093/jpepsy/jsab089

Knox, M., Burkhart, K., & Khuder, S.A. (2011). Parental hostility and depression as predictors of young children’s aggression and conduct problems. *Journal of Aggression, Maltreatment & Trauma, 20*, 800–811. doi:10.1080/10926771.2011.610772

Lin, G.-X., Mikolajczak, M., Keller, H., Akgun, E., Arikan, G., Aunola, K., Barham, E., Besson, E., Blanchard, M.A., Boujut, E., Brianda, M.E., Brytek-Matera, A., César, F., Chen, B.-B., Dorard, G., dos Santos Elias, L. C., Dunsmuir, S., Egorova, N., Escobar, M. J., … Roskam, I. (2023). Parenting culture(s): Ideal-parent beliefs across 37 countries. *Journal of Cross-Cultural Psychology, 54*, 4–24. https://doi.org/10.1177/00220221221123043

Lin, G. X., & Szczygieł, D. (2022). Perfectionistic parents are burnt out by hiding emotions from their children, but this effect is attenuated by emotional intelligence. *Personality and Individual Differences, 184*, 111187. https://doi.org/10.1016/j.paid.2021.111187

Lindström, C., Åman, J., Anderzén-Carlsson, A., & Lindahl Norberg, A. (2016). Group intervention for burnout in parents of chronically ill children - a small-scale study. *Scandinavian Journal of Caring Sciences*, *30*, 678–686. https://doi.org/10.1111/scs.12287

Marchetti, D., Fontanesi, L., Mazza, C., Di Giandomenico, S., Roma, P., & Verrocchio, M.C. (2020). Parenting-related exhaustion during the Italian COVID-19 lockdown. *Journal of Pediatric Psychology*, *45*, 1114–1123. https://doi.org/10.1093/jpepsy/jsaa093

Masarik, A.S., & Conger, R.D. (2017). Stress and child development: a review of the Family Stress Model. *Current Opinion in Psychology*, *13*, 85–90. https://doi.org/10.1016/j.copsyc.2016.05.008

Maslach, C., & Leiter, M.P. (2016). Understanding the burnout experience: Recent research and its implications for psychiatry. *World Psychiatry,* *15*, 103–111. https://doi.org/10.1002/wps.20311

Mikolajczak, M., Brianda, M.E., Avalosse, H., & Roskam, I. (2018). Consequences of parental burnout: Its specific effect on child neglect and violence. *Child Abuse & Neglect*, *80*, 134–145. https://doi.org/10.1016/j.chiabu.2018.03.025

Mikolajczak, M., Gross, J.J., & Roskam, I. (2019). Parental burnout: What is it, and why does it matter?. *Clinical Psychological Science*, *7*, 1319–1329. https://doi.org/10.1177/ 2167702619858430

Mikolajczak, M., Gross, J.J., & Roskam, I. (2021). Beyond job burnout: Parental burnout!. *Trends in Cognitive Sciences*, *25*, 333–336. https://doi.org/10.1016/j.tics.2021.01.012

Mikolajczak, M., Gross, J.J., Stinglhamber, F., Lindahl Norberg, A., & Roskam, I. (2020). Is parental burnout distinct from job burnout and depressive symptoms? *Clinical Psychological Science*, *8*, 673–689. https://doi.org/10.1177/2167702620917447

Mikolajczak, M., & Roskam, I. (2018). A theoretical and clinical framework for parental burnout: the balance between risks and resources (BR2). *Frontiers in Psychology*, *9*, 886. https://doi.org/10.3389/fpsyg.2018.00886

Mikolajczak, M., & Roskam, I. (2020). Parental burnout: Moving the focus from children to parents. *New Directions for Child and Adolescent Development*, *174*, 7–13. https://doi.org/10.1002/cad.20376

[hidden for blind review]

Nelson, J.A., Boyer, B.P., Villarreal, D.L., & Smith, O.A. (2017). Relations between mothers’ daily work, home, and relationship stress with characteristics of mother-child conflict interactions. *Journal of Family Psychology*, *31*, 431–441. https://doi.org/10.1037/fam0000276

Nezlek, J.B., Rusanowska, M., Holas, P., & Krejtz, I. (2021). The factor structure of a Polish language version of the hospital anxiety depression scale (HADS). Current Psychology*, 40*, 2318–2326. https://doi.org/10.1007/s12144-019-0164-0

Prikhidko, A., Long, H., & Wheaton, M.G. (2020). The effect of concerns about COVID-19 on anxiety, stress, parental burnout, and emotion regulation: The role of susceptibility to digital emotion contagion. *Frontiers in Public Health*, *8*, 567250. https://doi.org/10.3389/fpubh.2020.567250

Putnick, D.L., Bornstein, M.H., Lansford, J.E., Malone, P.S., Pastorelli, C., Skinner, A.T., Sorbring, E., Tapanya, S., Uribe Tirado, L.M., Zelli, A., Alampay, L.P., Al-Hassan, S.M., Bacchini, D., Bombi, A.S., Chang, L., Deater-Deckard, K., Di Giunta, L., Dodge, K.A., & Oburu, P. (2015). Perceived mother and father acceptance-rejection predict four unique aspects of child adjustment across nine countries. *Journal of Child Psychology and Psychiatry*, *56*, 923–932. https://doi.org/10.1111/jcpp.12366

Robinaugh, D.J., Hoekstra, R., Toner, E.R., & Borsboom, D. (2020). The network approach to psychopathology: a review of the literature 2008-2018 and an agenda for future research. *Psychological Medicine*, *50*, 353–366. https://doi.org/10.1017/S0033291719003404

Roskam, I., Brianda, M.E., & Mikolajczak, M. (2018). A step forward in the conceptualization and measurement of parental burnout: The parental burnout assessment (PBA). *Frontiers in Psychology*, *9*, Article 758. https://doi.org/10.3389/fpsyg.2018.00758.

Roskam, I., & Mikolajczak, M. (2020). Gender differences in the nature, antecedents and consequences of parental burnout. *Sex Roles, 83*, 485–498. https://doi.org/10.1007/s11199-020-01121-5

Roskam, I. & Mikolajczak, M. (2021). The slippery slope of parental exhaustion: A process model of parental burnout. *Journal of Applied Developmental Psychology, 77*, 101354. https://doi.org/10.1016/j.appdev.2021.101354

Roskam, I., Raes, M.E., & Mikolajczak, M. (2017). Exhausted parents: Development and preliminary validation of the Parental Burnout Inventory. *Frontiers in Psychology*, *8*, 163. https://doi.org/10.3389/fpsyg.2017.00163

Sawyer, S.M., Azzopardi, P.S., Wickremarathne, D., & Patton, G.C. (2018). The age of adolescence. *The Lancet. Child & Adolescent Health*, *2*, 223–228. https://doi.org/10.1016/S2352-4642(18)30022-1

Schellekens, M.P.J., Wolvers, M.D.J., Schroevers, M.J., Bootma, T.I., Cramer, A.O.J., & van der Lee, M.L. (2020). Exploring the interconnectedness of fatigue, depression, anxiety and potential risk and protective factors in cancer patients: a network approach. *Journal of Behavioral Medicine, 43*, 553–563. https://doi.org/10.1007/s10865-019-00084-7

Skjerdingstad, N., Johnson, M.S., Johnson, S.U., Hoffart, A., Ebrahimi, O.V. (2021). Feelings of worthlessness links depressive symptoms and parental stress: A network analysis during the COVID-19 pandemic. *European Psychiatry*, *64*, e50, 1–10 https://doi.org/10.1192/j.eurpsy.2021.2223

Somma, A., Krueger, R.F., Markon, K.E., Gialdi, G., Colanino, M., Ferlito, D., Liotta, C., Frau, C., & Fossati, A. (2021). A longitudinal study on clinically relevant self-reported depression, anxiety and acute stress features among Italian community-dwelling adults during the COVID-19 related lockdown: Evidence of a predictive role for baseline dysfunctional personality dimensions. *Journal of Affective Disorders,* *282*, 364–371. https://doi.org/10.1016/j.jad.2020.12.165

Stavridou, A., Stergiopoulou, A.A., Panagouli, E., Mesiris, G., Thirios, A., Mougiakos, T., Troupis, T., Psaltopoulou, T., Tsolia, M., Sergentanis, T.N., & Tsitsika, A. (2020). Psychosocial consequences of COVID-19 in children, adolescents and young adults: A systematic review. *Psychiatry and Clinical Neurosciences*, *74*, 615–616. https://doi.org/10.1111/pcn.13134

Stone, L.L., Mares, S. H., Otten, R., Engels, R.C., & Janssens, J.M. (2016). The co-development of parenting stress and childhood internalizing and externalizing problems. *Journal of Psychopathology and Behavioral Assessment, 38*, 76–86. https://doi.org/10.1007/s10862-015-9500-3

Szczygieł, D., Sekulowicz, M., Kwiatkowski, P., Roskam, I., & Mikolajczak, M. (2020). Validation of the Polish version of the Parental Burnout Assessment (PBA). *New Directions for Child and Adolescent Development*, 174, 137–158. https://doi.org/10.1002/cad.20385

Thomas, A.S., Osbourne, M., Appelhans, B.M., Roisman, G.I., Booth-LaForce, C., & Bleil, M.E. (2022). Disparities in COVID-19-related stressful life events in the United States: Understanding who is most impacted. *Health & Social Care in the Community, 30*, 1199–1211. https://doi.org/10.1111/hsc.13671

Vacaru, S.V., Beijers, R., & de Weerth, C. (2022). Internalizing symptoms and family functioning predict adolescent depressive symptoms during COVID-19: A longitudinal study in a community sample. *PLoS ONE, 17*, Article e0264962. https://doi.org/10.1371/journal.pone.0264962

van Bakel, H., Bastiaansen, C., Hall, R., Schwabe, I., Verspeek, E., Gross, J.J., Brandt, J.A., Aguiar, J., Akgun, E., Arikan, G., Aunola, K., Bajgarová, Z., Beyers, W., Bílková, Z., Boujut, E., Chen, B.-B., Dorard, G., Escobar, M.J., Furutani, K., … & Roskam, I. (2022). Parental burnout across the globe during the COVID-19 pandemic. *International Perspectives in Psychology: Research, Practice, Consultation, 11*, 141–152. https://doi.org/10.1027/2157-3891/a000050

Vermeulen, S., Alink, L., & van Berkel, S.R. (2022). Child maltreatment during school and childcare closure due to the COVID-19 pandemic. *Child Maltreatment*, 10775595211064885. Advance online publication. https://doi.org/10.1177/10775595211064885

Viner, R. M., Ozer, E.M., Denny, S., Marmot, M., Resnick, M., Fatusi, A., & Currie, C. (2012). Adolescence and the social determinants of health. *The Lancet*, *379*, 1641–1652. https://doi.org/10.1016/S0140-6736(12)60149-4

Wang, J., Wang, H., Lin, H., Richard, M., Yang, S., Liang, H., Chen, X., & Fu, C. (2021). Study problems and depressive symptoms in adolescents during the COVID-19 outbreak: poor parent-child relationship as a vulnerability. *Globalization and Health,* *17*,  Article 40. https://doi.org/10.1186/s12992-021-00693-5

Watrowski, R., & Rohde, A. (2014). Validation of the Polish version of the Hospital Anxiety and Depression Scale in three populations of gynecologic patients. *Archives of Medical Science, 10*, 517–524. https://doi.org/10.5114/aoms.2013.36520

Wołowicz-Ruszkowska, A. (2016). How Polish women with disabilities challenge the meaning of motherhood. *Psychology of Women Quarterly, 40*, 80–95. https://doi.org/10.1177/0361684315600390

Wu, M., Xu, W., Yao, Y., Zhang, L., Guo, L., Fan, J., & Chen, J. (2020). Mental health status of students' parents during COVID-19 pandemic and its influence factors. *General Psychiatry*, 33, e100250. https://doi.org/10.1136/gpsych-2020-100250

Yıldırım, M., Çiçek, İ., & Şanlı, M.E. (2021). Coronavirus stress and COVID-19 burnout among healthcare staffs: The mediating role of optimism and social connectedness. *Current Psychology, 40*, 5763–5771. https://doi.org/10.1007/s12144-021-01781-w

Yıldırım, M., & Solmaz, F. (2022). COVID-19 burnout, COVID-19 stress and resilience: Initial psychometric properties of COVID-19 Burnout Scale. *Death Studies*, *46*, 524–532. https://doi.org/10.1080/07481187.2020.1818885

Zhen, B., Yao, B., & Zhou, X. (2022). How does parent-child communication affects posttraumatic stress disorder and growth in adolescents during the COVID-19 pandemic? The mediating roles of self-compassion and disclosure. *Journal of Affective Disorders*, *306*, 1–8. https://doi.org/10.1016/j.jad.2022.03.029

Zigmond, A., & Snaith, R. (1983). The hospital anxiety and depression scale. *Acta Psychiatrica Scandinavica, 67*, 361–370.
