# Supplementary material for: Parental and Pandemic Burnout, Internalizing Symptoms, and Parent-Adolescent Relationships: A Network Analysis
Source: J Psychopathol Behav Assess. 2023 Mar 14;45(2):428–43. doi: 10.1007/s10862-023-10036-w (PMC10010964; doi:10.1007/s10862-023-10036-w)
Supplement: Supplementary file 2 — Supplementary Material 2 [file 10862_2023_10036_MOESM2_ESM.docx]

**Parental and pandemic burnout, internalizing symptoms, and parent-adolescent relationships: A network analysis**

**Supplementary materials**

**Figure S1**

*Simulation results using the estimated refitted network as true network structure. The panel shows the sensitivity (true positive rate), specificity (true negative rate) and correlation between true and estimated networks*


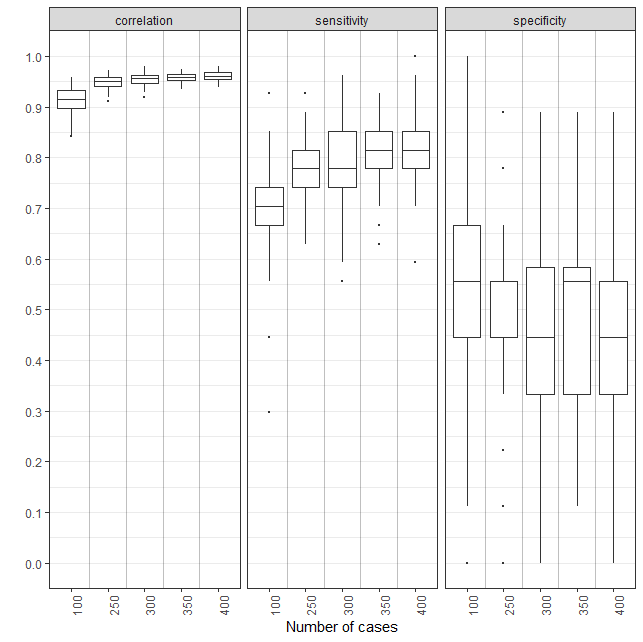


**Figure S2**

*Bootstrapped Confidence Intervals of Estimated Weights for the Graphical Gaussian Model*


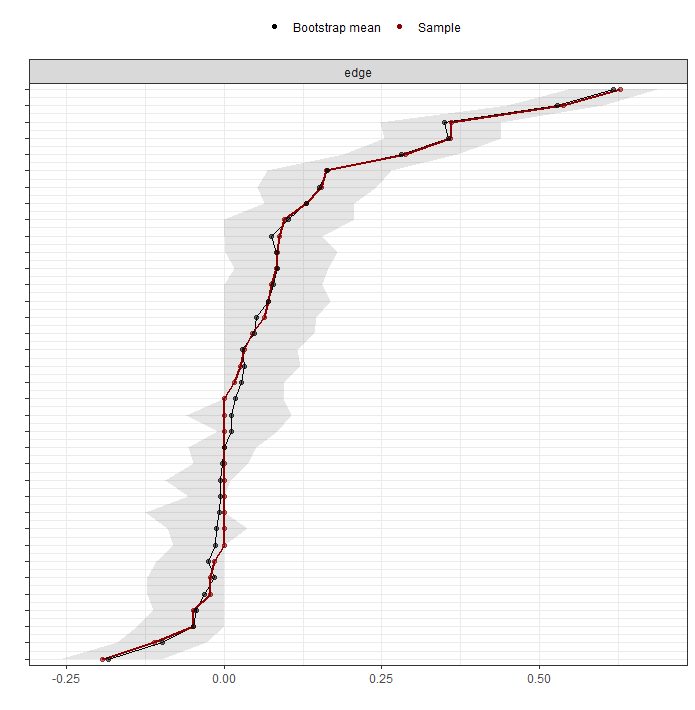


*Note.* The red line indicates the values from the original sample, while the dark line indicates the mean bootstrapped values. The gray area indicates the 95% confidence intervals.

**Figure S3**

*Bootstrapped Difference Tests (α = 0.05) Between Non-zero Edge Weights in the Estimated Network*


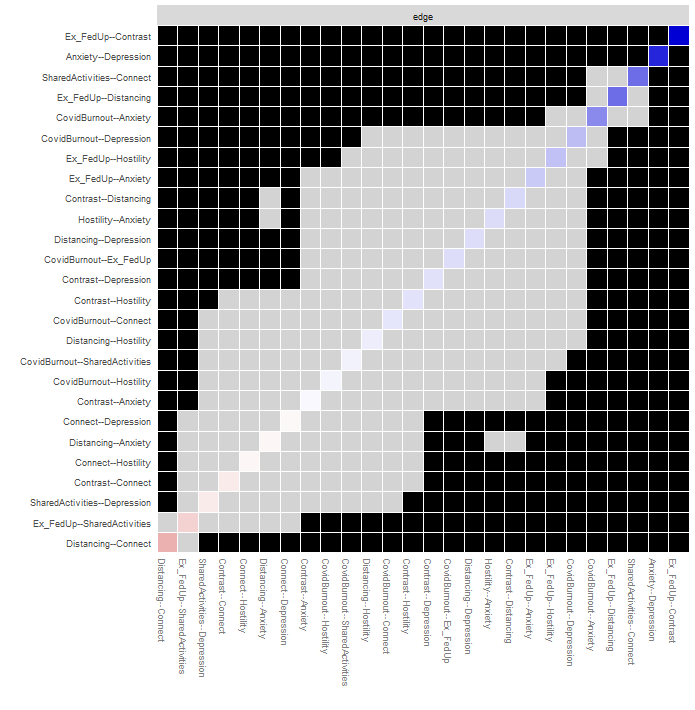


*Note*. Black boxes denote edges that significantly differ from one another, while gray boxes indicate edges that do not. Colored boxes indicate the weight of that edge, with a darker color indicating a larger weight. Ex_FedUp = Emotional exhaustion and feelings of being fed up; Contrast = Loss of parental accomplishment and efficacy.

**Figure S4**

*Average Correlations Between Centrality Indices (i.e., Expected Influence and Bridge Expected Influence) of the Original Network and Network with Persons Dropped*


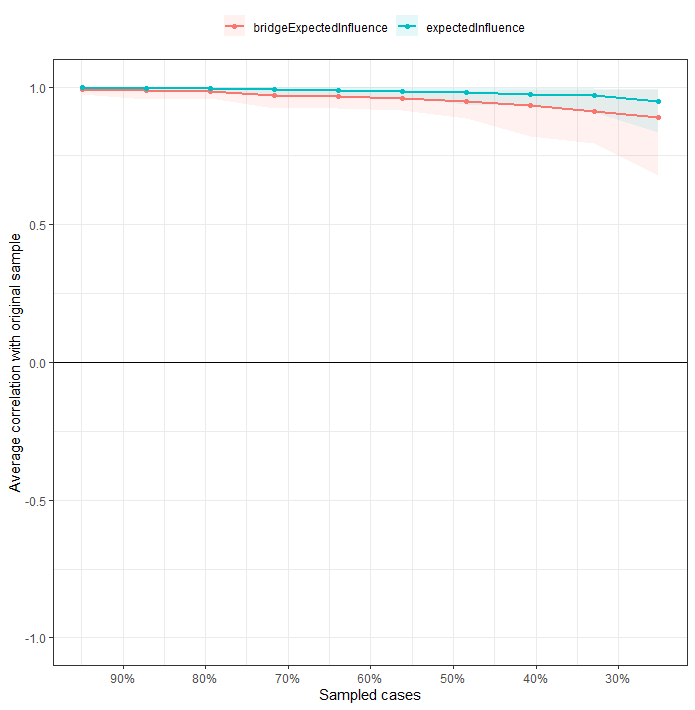


**Figure S5**

*Bootstrapped Difference Tests Between the Expected Influence of Nodes*


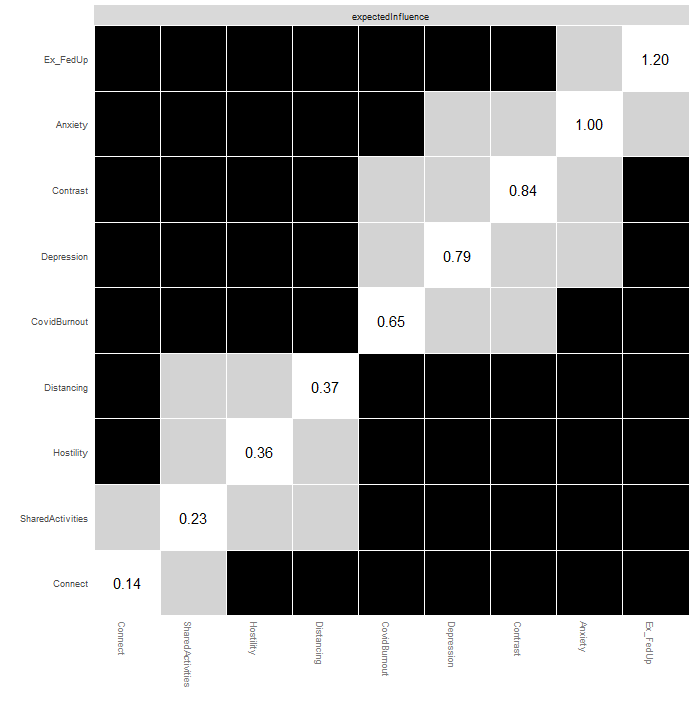


*Note.* Black boxes denote edges that significantly differ from one another, while gray boxes indicate edges that do not. Ex_FedUp = Emotional exhaustion and feelings of being fed up; Contrast = Loss of parental accomplishment and efficacy.

**Figure S6**

*Bootstrapped Difference Tests Between the Bridge Expected Influence of Nodes*


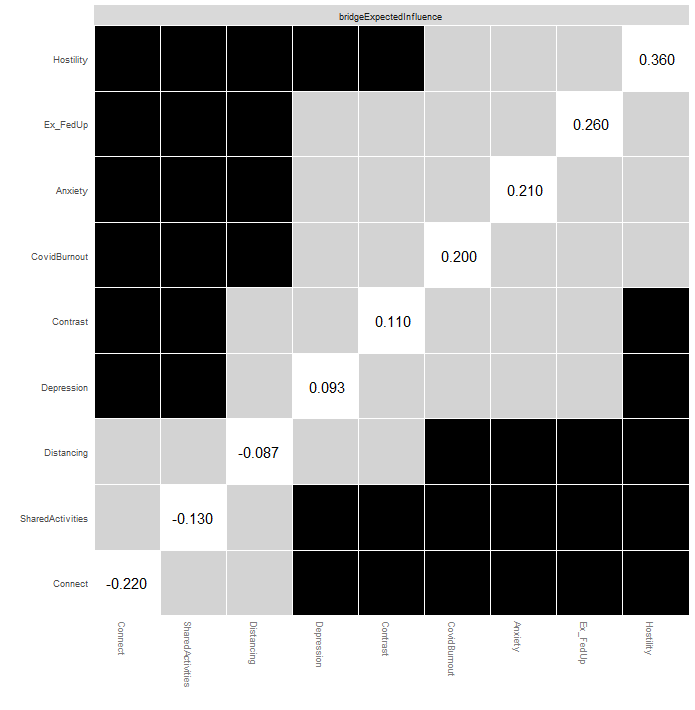


*Note.* Black boxes denote edges that significantly differ from one another, while gray boxes indicate edges that do not. Ex_FedUp = Emotional exhaustion and feelings of being fed up; Contrast = Loss of parental accomplishment and efficacy.

Table S1

*Bridge Expected Influence of Nodes Belonging to the Particular Communities*

| Node | Parental burnout ↔ Parent-adolescent relationship | Pandemic burnout/parental internalizing symptoms ↔ Parent-adolescent relationship | Parental burnout ↔ Pandemic burnout/parental internalizing symptoms |
| --- | --- | --- | --- |
| Exhaustion/FedUp | .038 |  | .247 |
| Distancing | -.194 |  | .110 |
| Contrast | .000 |  | .086 |
| Pandemic burnout |  | 0 | .096 |
| Depression |  | 0 | .196 |
| Anxiety |  | 0 | .152 |
| Connectedness | -.194 | 0 |  |
| Shared Activities | -.115 | 0 |  |
| Hostility | .153 | 0 |  |
